# Tight regulation of the unfolded protein sensor Ire1 by its intramolecularly antagonizing subdomain

**DOI:** 10.1242/jcs.164111

**Published:** 2015-05-01

**Authors:** Rubwad Mathuranyanon, Tomoko Tsukamoto, Asumi Takeuchi, Yuki Ishiwata-Kimata, Yuichi Tsuchiya, Kenji Kohno, Yukio Kimata

**Affiliations:** Graduate School of Biological Sciences, Nara Institute of Science and Technology, 8916-5 Takayama, Ikoma, Nara 630-0192, Japan

**Keywords:** Unfolded protein response, Stress response, Endoplasmic reticulum, Intrinsically disordered region, Molecular chaperone, Misfolded protein

## Abstract

Accumulation of unfolded proteins in the endoplasmic reticulum (ER) accompanies ER stress and causes the type-I transmembrane protein Ire1 (also known as ERN1) to trigger the unfolded protein response (UPR). When dimerized, the core stress-sensing region (CSSR) of Ire1 directly captures unfolded proteins and forms a high-order oligomer, leading to clustering and activation of Ire1. The CSSR is N-terminally flanked by an intrinsically disordered subdomain, which we previously named Subregion I, in *Saccharomyces cerevisiae* Ire1. In this study, we describe tight repression of Ire1 activity by Subregion I under conditions of no or weak stress. Weak hyperactivation of an Ire1 mutant lacking Subregion I slightly retarded growth of yeast cells cultured under unstressed conditions. Fungal Ire1 orthologs and the animal Ire1 family protein PERK (also known as EIF2AK3) carry N-terminal intrinsically disordered subdomains with a similar structure and function to that of Subregion I. Our observations presented here cumulatively indicate that Subregion I is captured by the CSSR as an unfolded protein substrate. This intramolecular subdomain interaction is likely to compromise self-association of the CSSR, explaining why Subregion I can suppress Ire1 activity when ER-accumulated unfolded proteins are not abundant.

## INTRODUCTION

The endoplasmic reticulum (ER) of eukaryotic cells is a cellular compartment where secretory and membrane proteins are folded. Impaired protein folding in the ER accompanies dysfunction of the ER, namely ER stress, and evokes the unfolded protein response (UPR). The UPR is a cellular protective event through which proteins in the ER are transcriptionally induced (Mori, 2009; Walter and Ron, 2011). Ire1 (also known as ERN1) is an ER-located type-I transmembrane endoribonuclease conserved among eukaryotic organisms, and it functions as an ER-stress sensor that triggers the UPR.

It is widely believed that Ire1 functions as a receptor for unfolded proteins accumulated in the ER (Credle et al., 2005; Kimata et al., 2007; Gardner and Walter, 2011). The luminal domain of Ire1 has a tightly folded region (Kimata et al., 2004; Credle et al., 2005; Oikawa et al., 2005) called the core stress-sensing region (CSSR; supplementary material Fig. S1A). According to the X-ray crystallographic analysis reported by Credle et al. (Credle et al., 2005), the dimeric form of CSSR has a deep groove that captures unfolded proteins. Gardner and Walter (Gardner and Walter, 2011) proposed that the CSSR is highly self-oligomerized when directly associated with unfolded proteins. This finding well explains the molecular mechanism by which Ire1 clusters during ER stress (supplementary material Fig. S1A; Kimata et al., 2007; Aragón et al., 2009; Li et al., 2010). According to X-ray crystallographic and biochemical analyses of the Ire1 cytosolic domain, clustered Ire1 molecules exhibit a potent RNA-cleaving activity (Korennykh et al., 2009).

In the case of *Saccharomyces cerevisiae* (hereafter called yeast), Ire1 performs splicing of the *HAC1* gene transcript (*HAC1*^i^) to yield the *HAC1*^u^ form, which is translated into a transcription factor that induces the UPR target genes (Cox and Walter, 1996). Cell growth is damaged when Ire1 is improperly activated or when the UPR is artificially induced (Mori et al., 2000; Chawla et al., 2011; Rubio et al., 2011), probably because the yeast cell transcriptome is drastically changed by the UPR (Travers et al., 2000; Kimata et al., 2006). This finding explains the reason for Ire1 being tightly regulated through additional mechanisms.

We and others have reported previously that Ire1 is negatively regulated by the ER-located molecular chaperone BiP (encoded by *KAR2*) (Bertolotti et al., 2000; Kimata et al., 2003; Kimata et al., 2004). The association of BiP with Ire1 is likely to inhibit the self-association of Ire1 in unstressed cells (Bertolotti et al., 2000), and ER stress causes dissociation of BiP from Ire1 (supplementary material Fig. S1A). The BiP-binding subdomain is located at the juxtamembrane position (Subregion V of yeast Ire1; Kimata et al., 2004) and is loosely folded (Oikawa et al., 2005). As BiP is induced by the UPR, the negative regulation of Ire1 by BiP is likely to be a feedback-control system (Pincus et al., 2010).

Metazoan cells also carry another Ire1-family ER-stress sensor called PERK (also known as EIF2AK3) that attenuates protein synthesis upon ER stress (Harding et al., 1999). Ire1 orthologs and PERK commonly possess juxtamembrane BiP-binding subdomains and highly conserved regions corresponding to the CSSR (supplementary material Fig. S1B; Liu et al., 2000; Kimata and Kohno, 2011). Thus, we believe that Ire1 orthologs and PERK are regulated and activated in a similar manner.

In addition, PERK and yeast Ire1, but not higher eukaryotic Ire1 orthologs, have unconserved subdomains at the N-terminus [N-terminal unconserved region (NUCR); supplementary material Fig. S1B]. The NUCR of yeast Ire1 is called Subregion I (supplementary material Fig. S1A,B; Kimata et al., 2004), and it appears to be intrinsically disordered, as Subregion I is susceptible to partial proteolysis (Oikawa et al., 2005). In the present study, we describe the physiological importance and molecular mechanism of tight negative regulation of Ire1 by the NUCR.

## RESULTS

### Subregion I suppresses yeast Ire1 activity

**Fig. 1.**
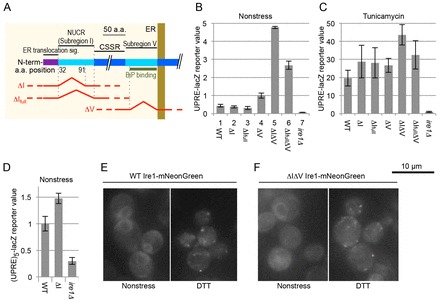
**Yeast Ire1 mutants carrying full-length or almost full-length deletions of Subregion I and/or Subregion V.** (A) The Ire1 mutations used in the experiments shown in panels B–D are illustrated. The ΔI, ΔI_full_ and ΔV mutations correspond to deletion of amino acids (a.a.) 32–91, amino acids 32–111 and amino acids 463–524 of yeast Ire1, respectively. The N-terminal (N-term) ER translocation signal is shown in purple. (B,C) After transformation with a single-copy *IRE1* plasmid [pRS313-IRE1; wild-type (WT)] or its mutants [or the empty vector pRS313 (*ire1Δ*)], KMY1015 *ire1Δ* cells carrying the UPRE-lacZ reporter plasmid (pCZY1) remained unstressed (B) or were stressed with 2 µg/ml tunicamycin for 4 h (C), and cellular β-galactosidase activity was measured and normalized to that of the unstressed Δ V-*IRE1* samples (set to 1.0). (D) A similar experiment to that shown in panel B was performed using *ire1Δ* KMY1516 [(UPRE)_5_-lacZ] cells. Data are normalized to that of the wild-type *IRE1* samples (set to 1.0). Data in B–D show the mean±s.d. (E,F) Yeast cells producing mNeonGreen-tagged Ire1 (wild-type Ire1–mNeonGreen) or its ΔIΔV mutant were subjected to fluorescence microscopy imaging.

At the beginning of this study, we checked some of the partial deletion mutants of yeast Ire1 (Fig. 1A) for their UPR-inducing ability using a UPRE-lacZ reporter gene that expresses β-galactosidase under control of the UPR-target promoter element (UPRE; Mori et al., 1992). In the experiment shown in Fig. 1B, cells were cultured under unstressed conditions before measuring cellular β-galactosidase activity. The UPR was slightly induced by the ΔV mutation, a full-length deletion of Subregion V (compare column 4 to 1), probably because this mutation abolishes the interaction between Ire1 and BiP.

The NUCR of yeast Ire1, namely Subregion I, is composed of 80 amino acid residues. In our previous studies (Oikawa et al., 2007; Kimata et al., 2007), we deleted a 60-amino-acid portion of Subregion I (hereafter called the Subregion I 60-amino-acid portion) to obtain the ΔI mutant. Reproducing our previous findings (Oikawa et al., 2007; Kimata et al., 2007), we observed that the ΔI mutation clearly activated Ire1 in unstressed cells when combined with the ΔV mutation (Fig. 1B, compare column 5 to columns 1, 2 and 4).

The ΔI_full_ mutation is a full-length deletion of Subregion I (Fig. 1A). The activity of ΔI_full_ΔV Ire1 was higher than that of ΔV Ire1, but lower than that of ΔIΔV Ire1 in unstressed cells (Fig. 1B, compare column 6 to columns 4 and 5). Thus, we employed the ΔI mutation, but not the ΔI_full_ mutation, in the subsequent experiments to determine the Ire1-suppressing ability of Subregion I. As shown in Fig. 1C, all Ire1 mutants used were considerably activated by treating cells with tunicamycin, which inhibits N-glycosylation and induces ER stress (note that the *y*-axis scale value of Fig. 1C is 10-fold larger than that of Fig. 1B).

Does the ΔI single mutation exhibit any apparent phenotype? The UPRE-lacZ reporter assay did not reveal a difference in activity between wild-type Ire1 and ΔI Ire1 (Fig. 1B, compare lane 2 to 1). We then employed another highly sensitive version of the UPR reporter assay in which expression of the lacZ gene was controlled under five tandem copies of the UPRE [(UPRE)_5_-LacZ; Promlek et al., 2011]. This method allowed us to monitor weak induction of the UPR at high resolution and to observe slightly higher ΔI-Ire1 activity than that of wild-type Ire1 in unstressed cells (Fig. 1D). As shown in supplementary material Fig. S2A, the ΔI or the ΔV mutations did not considerably affect cellular abundance of Ire1.

According to Shaner et al. (Shaner et al., 2013), mNeonGreen is a bright green fluorescent protein that works as a superior substitute for the *Aequorea* GFP derivatives. In the experiments shown in Fig. 1E,F, mNeonGreen-tagged Ire1 or its ΔIΔV mutant version was expressed from a single-copy plasmid under control of the authentic *IRE1* promoter. Both wild-type Ire1 and ΔIΔV Ire1 distributed diffusely, probably on the ER, under unstressed conditions, whereas they exhibited dot-like distribution in response to ER stress caused by dithiothreitol (DTT; a disulfide reducing agent). This finding suggests that neither Subregion I nor V is involved in the cluster-formation step of Ire1. It should be noted that our observation presented here is inconsistent with our previous report in which ΔIΔV Ire1 constitutively clustered even under unstressed conditions when artificially overexpressed from a multicopy plasmid (Kimata et al., 2007).

**Fig. 2.**
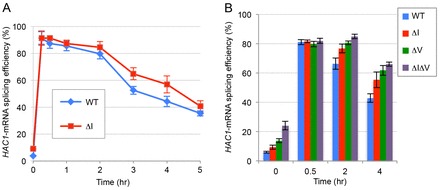
**Timecourse profile of wild-type, ΔI, ΔV and ΔIΔV Ire1 activity.**-copy *IRE1* plasmid [pRS313-IRE1; wild-type (WT)] or its mutants, KMY1516 *ire1Δ* cells were stressed with 3 mM DTT for the indicated times. *HAC1* mRNAs were then amplified by RT-PCR from total RNA samples to obtain the *HAC1* mRNA splicing efficiency values, which are expressed as the mean±s.d. from three independent transformant clones.

Next, we directly checked the splicing of *HAC1* mRNA to compare activities of wild-type Ire1 and ΔI Ire1 in cells (Fig. 2A). Supporting the result shown in Fig. 1D, the ΔI single mutation weakly enhanced *HAC1* mRNA splicing under unstressed conditions (Time 0). Moreover, whereas UPR activation profiles following treatment with 3 mM DTT were almost equal in cells expressing wild-type *IRE1* or the ΔI mutant at the early time-points (15 min to 1 h), attenuation of *HAC1* mRNA splicing under long-term stress (2–5 h) appeared to be slightly retarded by the ΔI mutation.

We then performed the same experiment as that shown in Fig. 2A using not only wild-type *IRE1* and ΔI-*IRE1* cells but also ΔV-*IRE1* and ΔIΔV-*IRE1* cells (Fig. 2B). As reported previously (Pincus et al., 2010; Ishiwata-Kimata et al., 2013), the ΔV mutation also caused an impaired attenuation of Ire1 activity upon prolonged ER stress. The *HAC1* mRNA splicing assay, as well as the UPRE-lacZ reporter assay shown in Fig. 1, demonstrated an aggravated hyperactivation of Ire1 resulting from the combination of the ΔI and the ΔV mutations.

In the experiment shown in Table 1, we checked whether the ΔI mutation affects cellular growth under unstressed conditions. Cells expressing wild-type *IRE1* and those expressing ΔI-*IRE1* were mixed and cultured for a long duration to observe subtle differences in the growth rate between the two cell lines. Then, their relative abundance was monitored, which indicated that the ΔI mutation retarded cellular growth. The ΔV and the ΔIΔV mutations also caused growth retardation.

### Primary structural properties of Subregion I

The UPRE-lacZ reporter values of ΔV-*IRE1* and ΔIΔV-*IRE1* cells under unstressed conditions were considerably different (Fig. 1B), allowing us to perform quick and high-resolution monitoring of the Ire1-suppressing ability of Subregion I and its mutants. Thus, we modified ΔV Ire1 by introducing various mutations into its Subregion I, and we tested for its activity to induce UPRE-lacZ reporter in unstressed cells to address the primary structural requirements of Subregion I for the Ire1-suppressing ability.

**Fig. 3.**
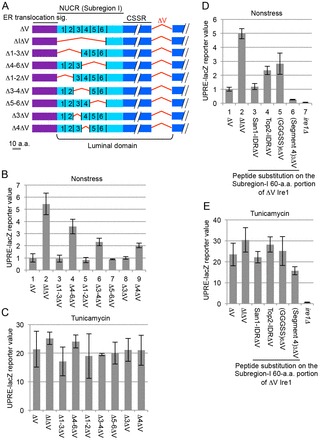
**Partial deletion and substitution mutations of Subregion I.**(A) The Subregion I 60-amino-acid (a.a.) portion (amino acids 32–91) was partitioned into six 10-amino-acid segments (Segments 1–6), which were serially deleted from ΔV Ire1 as illustrated. (B,C) A similar experiment and data presentation to that shown in Fig. 1B,C was performed using the Ire1 mutants shown in panel A. (D,E) A similar experiment and data presentation to that shown in Fig. 1B,C was performed using the ΔV Ire1 variants on which the Subregion I 60-amino-acid portion was replaced with San1 IDR, Top2 IDR, a six tandem repeat of the 5-amino-acid peptide GGGSS or the three tandem repeat of Segment 4. Data in B–E show the mean±s.d.

As illustrated in Fig. 3A, the Subregion I 60-amino-acid portion was partitioned into six 10-amino-acid segments (Segments 1–6; see supplemental material Table S1), which were serially deleted from ΔV Ire1 (refer to supplementary material Table S2 for the resulting amino acid sequences). We observed that ΔV Ire1 was not as highly activated as ΔIΔV Ire1 in unstressed cells even when carrying any of the partial deletions (Fig. 3B). Nevertheless, Fig. 3B also shows that the Δ4–6, Δ3–4 and Δ4 mutations, but not the Δ1–3, Δ1–2, Δ5–6 or Δ3 mutations, partially activated ΔV Ire1. Therefore, no specific Subregion I sequence was absolutely required for the Ire1-suppressing ability, whereas Segment 4 appears to be relatively important. All Ire1 mutants responded well to ER stress induced by tunicamycin (Fig. 3C). Moreover, these partial deletion mutations did not considerably affect cellular abundance of ΔV Ire1 (supplementary material Fig. S2B).

Two of our previous reports (Kimata et al., 2004; Oikawa et al., 2005) and a web-based computer prediction (supplementary material Fig. S3A; Ishida and Kinoshita, 2007) strongly suggest that Subregion I is intrinsically disordered. We next replaced the Subregion I 60-amino-acid portion of ΔV Ire1 with other 60-amino-acid sequences to determine whether intrinsically disordered unrelated peptides could work as Subregion I (refer to supplementary material Table S3 for amino acid sequences of the peptides). As shown in Fig. 3D, the intrinsically disordered region (IDR) of the unrelated protein San1 (Rosenbaum et al., 2011) suppressed Ire1 activity in unstressed cells when substituted for Subregion I (compare column 3 to columns 1 and 2). Moreover, the IDR of Top2 (Berger et al., 1996) and a six tandem repeat of a 5-amino-acid peptide GGGSS also exhibited Ire1-suppressing ability (columns 4 and 5), although less potently. Notably, a three tandem repeat of Segment 4 had stronger Ire1-suppressing ability than that of the authentic Subregion I (compare columns 6 and 1). (Segment 4)_3_ΔV Ire1, as well as the other Ire1 mutants employed here, responded well to tunicamycin-induced ER stress (Fig. 3E). Moreover, the cellular abundance of (Segment 4)_3_ΔV Ire1 did not differ from that of the other Ire1 mutants (supplementary material Fig. S2C).

### Subregion I is replaceable by the NUCRs of fungal Ire1 and mammalian PERK

**Fig. 4.**
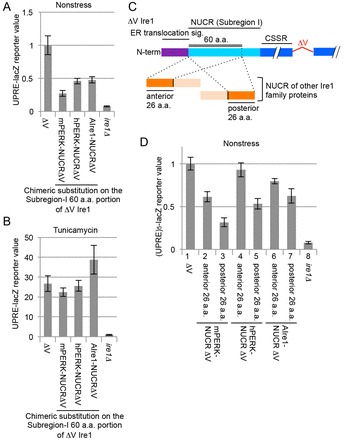
**Replacement of yeast Ire1 Subregion I with the NUCRs of other IRE1 family proteins.**(A,B) A similar experiment and data presentation to that shown in Fig. 1B,C was performed using the ΔV Ire1 variants on which the Subregion I 60-amino-acid (a.a.) portion was chimerically replaced with the NUCRs of mouse PERK (mPERK), human PERK (hPERK) or *Aspergillus oryzae* Ire1 (AIre1). (C) As illustrated, the NUCRs of the other IRE1 family proteins (orange colored) were divided into anterior and posterior portions and substituted for the Subregion I 60-amino-acid portion of ΔV Ire1. (D) A similar experiment to that shown in Fig. 1D was performed using the Ire1 mutants shown in panel C. Data are normalized to that of the ΔV-*IRE1* samples (set to 1.0). Data in A,B,D show the mean±s.d.

We then asked whether the NUCRs of the fungal Ire1 ortholog and mammalian PERK, which are predicted to be intrinsically disordered (supplementary material Fig. S3B), also have the Subregion-I-like Ire1-suppressing ability. Thus, we constructed yeast ΔV Ire1 chimeric mutants in which the Subregion I 60-amino-acid portion was replaced with similar-length (52-amino-acid) NUCR sequences derived from mammalian PERK or *Aspergillus oryzae* Ire1 (refer to supplementary material Table S3 for the amino acid sequences). According to the UPRE-lacZ reporter assay results shown in Fig. 4A, the chimeric constructs were less active than ΔV Ire1, which carried the authentic Subregion I, in unstressed cells, whereas they responded well to ER stress (Fig. 4B). These chimeric mutations did not considerably affect cellular abundance of ΔV Ire1 (supplementary material Fig. S2C,D). This observation strongly suggests a potent function of the NUCRs in suppressing Ire1 activity.

Next, the 52-amino-acid NUCR sequences were partitioned into two half-length segments, which were substituted for the Subregion I 60-amino-acid portion of ΔV Ire1 (Fig. 4C; see supplemental material Table S3). We then observed that, in unstressed cells, the posterior segments commonly suppressed Ire1 function to a greater extent than the anterior segments did (Fig. 4D, compare column 3 to 2, 5 to 4 and 7 to 6). Notably, the posterior 30-amino-acid segment had an Ire1-suppressing ability that was greater than that of the anterior 30-amino-acid segment also in the case of authentic yeast Ire1 Subregion I (Fig. 3B, compare column 3 to 4). Thus, we think that the NUCRs have a common structural feature that cannot be predicted from a simple sequence comparison.

**Fig. 5.**
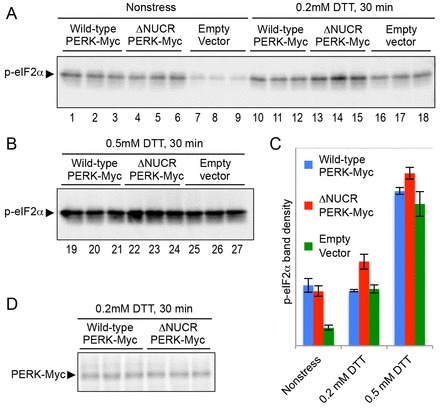
**Hyperactivation of murine PERK by its NUCR deletion mutation.**(A,B,D) After transfection of pcDNAmPERK-Myc (wild-type PERK–Myc overexpression), its ΔNUCR mutant or the empty vector pCDNA3.1(+), NIH3T3 cells were cultured for 24 h and stressed as indicated. Cell lysates were then analyzed by western blot analysis with anti-phospho-eIF2α (A,B) or anti-Myc (D). Three independent samples prepared under identical conditions were analyzed. (C) Band densities of panels A and B were measured and are expressed relative to that of the nonstressed empty-vector samples. Data show the mean±s.d. (*n* = 3).

We then asked whether mammalian PERK is hyperactivated by the deletion mutation of its NUCR. In the experiment shown in Fig. 5, murine PERK-expression plasmids were transfected into a murine cultured cell line, NIH3T3. The pcDNAmPERK-Myc (Harding et al., 1999) was used for overexpression of Myc epitope-tagged murine PERK (PERK–Myc) under control of the strong CMV promoter. We also employed a truncated version of this plasmid for expression of a mutant form of PERK–Myc not carrying NUCR (the ΔNUCR mutation). We then monitored phosphorylation of eIF2α (also known as EIF2S1), which is the direct phosphorylation target of PERK. As shown in Fig. 5A,C, the phosphorylation level of eIF2α seemed to be equally induced by transfection of either the PERK–Myc plasmid or its ΔNUCR version (compare lanes 1–6 to 7–9). Probably because of the endogenous PERK protein, DTT treatment induced eIF2α phosphorylation even in the case of the empty-vector-transfected cells (Fig. 5A–C, compare lanes 16–18 and 25–27 to 7–9). Importantly, as compared with the wild-type PERK–Myc-transfected or the empty-vector-transfected cells, cells expressing the ΔNUCR mutant version of PERK–Myc exhibited a higher-level phosphorylation of eIF2α upon weak ER stress induced by 0.2 mM DTT (Fig. 5A,C, compare lanes 13–15 to 10–12 and 16–18). A similar tendency, although less pronounced, was observed when cells were treated with 0.5 mM DTT (Fig. 5B,C, compare lanes 22–24 to 19–21 and 25–27). The anti-Myc western blot analysis of cell lysates shown in Fig. 5D indicates that wild-type PERK–Myc and its ΔNUCR version were expressed at similar levels in this transfection experiment.

### The Ire1-suppressing ability of Subregion I likely results from its intramolecular interaction with the CSSR

**Fig. 6.**
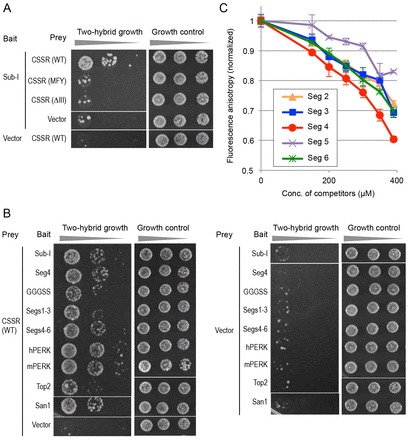
**Two-hybrid and *in vitro* interaction of the CSSR with various peptides.** (A,B) A yeast two-hybrid analysis was performed using the CSSR and its mutants as prey. The Subregion I 60-amino-acid portion (Sub-I), Segment 4 (Seg4; six tandem repeat), GGGSS (12 tandem repeat), Segments 1–3 (Segs1–3; amino acids 32–61 of yeast Ire1; two tandem repeat), Segments 4–6 (Segs4–6; amino acids 62–91 of yeast Ire1; two tandem repeat), the NUCR of human or mouse PERK (hPERK or mPERK), the Top2 IDR (Top2; two tandem repeat) and the San1 IDR (San1) were used as bait. Tester cell cultures were serially diluted tenfold, spotted on agar plates, incubated for 2–4 days and photographed. WT, wild type. (C) A fluorescently labeled peptide DEspP–FAM (10 µM final concentration) was mixed with MBP–CSSR (5 µM final concentration) and a competitor peptide [Segment (Seg) 2–6] and measured for fluorescence anisotropy. The resulting values from triplicate assays (mean±s.d.) are normalized to that for the no competitor peptide condition.

Because no specific primary structure of Subregion I appeared to be absolutely required for the Ire1-suppressing ability, we hypothesized that the CSSR, which captures substrate peptides rather nonspecifically, was involved (Gardner and Walter, 2011). Thus, we determined whether Subregion I could be a CSSR substrate. In the experiment shown in Fig. 6A, the Gal4 DNA-binding domain was fused to the Subregion I 60-amino-acid portion and used as bait in a yeast two-hybrid assay (see supplementary material Table S4 for the amino acid sequence). To yield prey, the Gal4 activation domain was fused to a CSSR peptide or that carrying the ΔIII or M229A/F285A/Y301A (MFY) mutation, which impairs the ability of the CSSR to capture substrate peptides (supplementary material Table S5; Kimata et al., 2007; Gardner and Walter, 2011; Promlek et al., 2011). Because the tester cells carry the *AUR1*-C gene controlled under a Gal4-inducible promoter, they acquire resistance to aureobasidin A when the two-hybrid system works. Subregion I and the CSSR exhibited a weak two-hybrid interaction, which, as expected, was impaired by the CSSR mutations.

As demonstrated in Fig. 6B, the CSSR also captured various peptides that appeared in the experiments shown in Figs 3, 4 (see supplementary material Table S4 for the amino acid sequences). In the two-hybrid analysis shown in Fig. 6B, the length of all the bait peptides was approximately 60 amino acids beause the short peptides were tandemly repeated. Considering colony appearance and size on the aureobasidin A plate, the two-hybrid interaction of the GGGSS repeat, the Top2 IDR and the Segment 1–3 peptides with the CSSR was weaker than that of the other peptides. This observation correlates well with our aforementioned finding that the Ire1-suppressing ability of the Top2 IDR, the (GGGSS)_6_ and the Segment 1–3 (Δ4–6) peptides was weaker than that of the San1 IDR, the (Segment 4)_3_, the Segment 4–6 (Δ1–3), the mPERK NUCR and the hPERK peptides (Figs 3, 4).

Next, we performed an *in vitro* competition assay to monitor the affinity of the Subregion I segments for the CSSR. A recombinant CSSR protein tagged with the maltose-binding protein (MBP–CSSR) was expressed in *Escherichia coli* and purified with amylose resin (Kimata et al., 2007). As originally described by Gardner and Walter (Gardner and Walter, 2011), a fluorescently labeled CSSR substrate, ΔEspP–FAM, exhibited increased fluorescence anisotropy when it was mixed with MBP–CSSR (supplementary material Fig. S4). Then, fluorescence anisotropy of ΔEspP–FAM was measured in the presence of MBP–CSSR and a chemically synthesized peptide carrying one of the Subregion I segment sequences (see supplementary material Table S6 for the amino acid sequences), indicating that Segment 4 compromises fluorescence anisotropy (by reducing the association between ΔEspP–FAM and MBP–CSSR), more effectively than the other segments did (Fig. 6C). This finding strongly suggests high-affinity capture of Segment 4 by the CSSR.

**Fig. 7.**
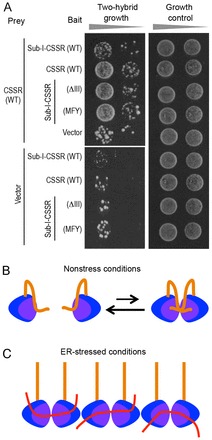
**Subregion I inhibits self-association of the CSSR.**(A) A yeast two-hybrid analysis was performed using the indicated peptide sequences as prey and bait. Subregion I-CSSR (Sub-I-CSSR) is a yeast Ire1 fragment carrying Subregion I and the CSSR (amino acids 33–454). WT, wild type. (B,C) Our proposed model for the function of Subregion I in Ire1 regulation is presented schematically. Under nonstress conditions, the groove-like structure (purple) of the CSSR (blue) captures Subregion I (orange), keeping the CSSR molecules monomeric (B). The CSSR forms a high-order oligomer when unfolded proteins instead of Subregion I are intermolecularly captured by the groove-like structure (C).

In the experiment shown in Fig. 7A, we used the yeast two-hybrid assay to monitor self-association of the CSSR (supplementary material Tables S4, S5). A two-hybrid interaction was observed when the CSSR was employed both as bait and prey. However, adding Subregion I to the bait CSSR (Subregion I-CSSR) abolished this two-hybrid interaction, which was restored by the ΔIII or the MFY mutation of Subregion I-CSSR. This finding strongly suggests that Subregion I leads to dissociation of the self-associated CSSR molecules when intramolecularly captured by the CSSR as a substrate.

## DISCUSSION

Growth of yeast cells is severely retarded by deregulated expression of the spliced form of *HAC1* mRNA (Mori et al., 2000). Moreover, according to Chawla et al. (Chawla et al., 2011) and Rubio et al. (Rubio et al., 2011), cells are damaged when Ire1 activity fails to be attenuated during long-term ER stress. Based on activation profiles of wild-type Ire1 and ΔI Ire1 (Fig. 1D; Fig. 2A), it is likely that Subregion I contributes to suppression of Ire1 activity under unstressed conditions and long-term ER stress. Although the difference in activity between wild-type Ire1 and ΔI Ire1 was not drastic, we think that Subregion I is beneficial for long-term proliferation of cells over many generations. Indeed, wild-type *IRE1* cells showed higher levels of proliferation than ΔI-*IRE1* cells in mixed cultures (Table 1). As shown in Fig. 5A,C, it is likely that the deletion of the NUCR also causes hyperactivation of murine PERK, especially under the weak ER stress condition.

Activation of Ire1 by the ΔI mutation was more obvious when it was combined with the ΔV mutation (Fig. 1B; Oikawa et al., 2007). Thus, we think that Subregion I and Subregion V suppress Ire1 activity in complementary fashions. Unlike the ΔV mutation, the ΔI mutation does not compromise the interaction between Ire1 and BiP (Oikawa et al., 2007). We thus think that Subregion I functions by a different mechanism in which BiP is not involved, whereas Subregion V serves as the BiP-binding site (Kimata et al., 2004).

The CSSR is likely to capture Subregion I, which is intrinsically disordered, as an unfolded protein substrate, because their two-hybrid interaction was abolished by the ΔIII or the MFY mutation (Fig. 6A). Our findings shown in Figs 3 and 4 indicate that various intrinsically disordered peptides exhibited potent or weak Ire1-suppressing ability when substituted for Subregion I. Peptides showing potent Ire1-suppressing ability commonly exhibited two-hybrid interactions with the CSSR that were stronger than those of peptides only weakly suppressing Ire1 (Fig. 6B). According to the *in vitro* competition analysis shown in Fig. 6C, the CSSR captured Segment 4, which is relatively important for the Ire1-suppressing ability of Subregion I (Fig. 3B), with a higher affinity than for other segments. These findings strongly suggest an intramolecular interaction between the CSSR and Subregion I (or peptides substituted for Subregion I) that suppresses Ire1 activity in unstressed cells. This interaction is likely to compromise self-association of the CSSR (Fig. 7A).

As shown in Fig. 7B, we thus propose a role for Subregion I as an intramolecular antagonist of the CSSR. We speculate that, under unstressed conditions, Subregion I is captured by the CSSR as an unfolded protein substrate and covers the dimerization interface of the CSSR, self-association of which is then inhibited. It is also possible that the dimeric form of the CSSR is dissociated through the intramolecular interaction of Subregion I with the CSSR. According to our model presented here, Subregion I and unfolded proteins compete for association with the CSSR. Therefore, instead of the intramolecular interaction of the CSSR with Subregion I, unfolded proteins are intermolecularly captured by the CSSR, which then forms the high-order oligomer (Gardner and Walter, 2011) under ER-stress conditions (Fig. 7C). This mechanism is highly unusual, as a receptor protein is carrying an antagonizing sequence.

The BiP–Ire1 association is also likely to contribute to maintaining Ire1 as a non-self-associated form (Bertolotti et al., 2000). Therefore, we think that Subregions I and V function as ‘double locks’ that compromise self-association of Ire1 under unstressed conditions. In the absence of this double lock, Ire1 remains as a dimer, as ΔIΔV Ire1 was self-associated constitutively but clustered in response to ER stress (Fig. 1E,F; Oikawa et al., 2007). According to Gardner and Walter (Gardner and Walter, 2011), the intermolecular interaction between unfolded proteins and dimeric CSSR molecules leads to the formation of the Ire1 cluster.

In the present paper, we propose that the NUCR portions of the Ire1 family proteins, which commonly show high disorder probability values in the PrDOS analysis (supplementary material Fig. S3), are captured by the CSSR as disordered peptides to suppress Ire1 activity. By contrast, it also should be noted that the disorder probability values of a peptide do not always correlate with its ability to be captured by the CSSR and to suppress Ire1 activity. For instance, the disorder probability of Segment 4 is not particularly high compared with those of the other segments (supplementary material Fig. S3A). According to our data shown in Fig. 4D, the posterior-half segments of the NUCR portions of the Ire1 family proteins commonly suppressed Ire1 function to a greater extent than the anterior-half segments did. In agreement with this observation, the posterior-half segments of the NUCR portions of mouse and human PERK show disorder probability values that are higher than those of the anterior-half segments; *A. oryzae* Ire1, however, does not show such a tendency (supplementary material Fig. S3B).

PERK, but not mammalian Ire1 orthologs (hereafter called IRE1), carries the NUCR (supplementary material Fig. S1B), which exhibited Ire1- or PERK-suppressing ability (Figs 4, 5). We thus speculate that, in mammalian cells, PERK might be inactivated more tightly than IRE1 under conditions of no or weak ER stress. Given that PERK inhibits global protein synthesis, it sounds reasonable that there exists a molecular machinery that suppresses PERK activity in healthy cells. By contrast, IRE1 functions in various physiological situations in development and homeostasis maintenance in mammals without external stress stimuli (Iwawaki et al., 2009; Iwawaki et al., 2010; Tsuru et al., 2013). As the internal physiological stress stimuli that are sensed by IRE1 might not be potent, we speculate that excessively tight suppression of IRE1 might be unfavorable for mammals. Indeed, according to Ma et al. (Ma et al., 2010), IRE1 but not PERK is activated upon differentiation of mature B cells to plasma cells, which secrete a large amount of antibody. We think that this idea might explain why the higher eukaryotic IRE1 does not carry the NUCR.

## MATERIALS AND METHODS

### Yeast cultures and strains

Unless otherwise noted, yeast cells were grown exponentially at 30°C under liquid-shaking culture in synthetic dextrose medium (2% glucose, 0.66% Difco yeast nitrogen base without amino acids, appropriate auxotrophic requirements). See Mori et al. (Mori et al., 1996), Kimata et al. (Kimata et al., 2004) and Promlek et al. (Promlek et al., 2011) for congenic haploid strains KMY1015 (*MATα leu2-3,112 ura3-52 his3-Δ200 trp1-Δ 901 lys2-801 ire1Δ::TRP1*) and KMY1516 [*MATα LEU2::*UPER-GFP*::leu2-3,112 ura3-52 his3-Δ200 trp1-Δ901 LYS2::*(UPRE)_5_-lacZ*::lys2-801 ire1Δ::TRP1*]. KMY1015 is a generous gift from Dr Kazutoshi Mori (Kyoto University, Japan). The *MATa* haploid strain Y2HGold and the *MATα* haploid strain Y187 were obtained from a commercial yeast two-hybrid system kit (Matchmaker Gold; Clontech).

### Plasmids

The yeast 2-μm plasmid pCZY1, which is a generous gift from Dr Kazutoshi Mori (Kyoto University, Japan), carries a UPRE-lacZ reporter construct (Mori et al., 1992). The yeast *IRE1* gene carrying the authentic 5′- and 3′-untranslated regions was previously cloned into the yeast single-copy vector pRS313 (Sikorski and Hieter, 1989) to obtain plasmid pRS313-IRE1 (Kimata et al., 2004). Partial deletion mutations were introduced into the *IRE1* gene on pRS313-IRE1, as described previously (Kimata et al., 2004), using the overlap PCR and *in vivo* homologous recombination techniques. We inserted the *Spe*I restriction sequence (5′-ACTAGT-3′) and the *Mlu*I restriction sequence (5′-ACGCGT-3′), respectively, after nucleotide position 93 and before nucleotide position 274 on the *IRE1* gene to introduce Subregion I substitution mutations into pRS313-IRE1, and then DNA fragments corresponding to the peptides shown in Figs 3 and 4 were placed in-frame between these two restriction sites. The San1 IDR (amino acids 339–394) and Top2 IDR (amino acids 634–659) were modified to carry the *Spe*I restriction or *Mlu*I restriction sequence (2 amino acids long each) on each end using the PCR technique. The NUCR fragments correspond to amino acids 29–80 of mouse PERK, amino acids 31–82 of human PERK and amino acids 28–79 of *Aspergillus oryzae* Ire1 and also carry the artificially attached *Spe*I restriction or *Mlu*I restriction sequence on each end. See supplementary material Table S3 for amino acid sequences of the Subregion I substitution mutants.

We used the pGBKT7 bait vector and the pGADT7 prey vector, both of which were obtained from the Matchmaker Gold kit (Clontech), for the two-hybrid analysis. The *IRE1* gene fragment corresponding to the CSSR (nucleotide positions 334–1362) or its mutants were cloned into the *Bam*HI/*Xho*I sites of pGADT7. Gene fragments of *IRE1* and its mutants were cloned into the *Nco*I/*Bam*HI sites of pGBKT7 for the experiment shown in Fig. 7A. DNA fragments corresponding to the bait peptides were cloned into the *Eco*RI/*Bam*HI sites of pGBKT7 for the experiments shown in Fig. 6A,B. See supplementary material Tables S4 and S5 for amino acid sequences of the bait and the prey peptides.

For overexpression of PERK–Myc, we used a mammalian expression plasmid pcDNA mPERK-Myc (Harding et al., 1999). As the empty vector control, pCDNA3.1(+) (Life Technologies) was employed. In order to generate the ΔNUCR mutant (deletion of amino acids 29–99) version of pcDNA mPERK-Myc, we performed PCR amplification of this plasmid with the PCR primer set 5′-TCCTTGGTAATCATCAGCACTTTAGATGGA-3′ and 5′-CGCAGAGATCCCCGCCGCGCAGCCCAGCAG-3′, the product of which was phosphorylated by T4 polynucleotide kinase and self-ligated.

### Genotypic analysis of yeast cells in mixed culture

A mixed culture of cells expressing wild-type *IRE1* (KMY1516 transformed with pRS313-IRE1) and cells expressing ΔI-*IRE1* (KMY1516 transformed with the ΔI-mutant version of pRS313-IRE1) was diluted and plated on agar plates to obtain isolated colonies. We then checked 100 colonies for their *IRE1* genotypes by PCR with the primer set 5′-CCATTATCACTTTTCTCCATATCA-3′ and 5′-GCAATTCTAAAATCTAAATCGCTT-3′. The abundance of ΔI-*IRE1* cells relative to wild-type cells (%) was calculated using the formula 100×[the number of ΔI-*IRE1* colonies/(the number of ΔI-*IRE1* colonies+the number of wild-type *IRE1* colonies)].

The same method was used to obtain the relative abundance of ΔIΔV-*IRE1* cells (%) from mixed cultures of cells expressing wild-type *IRE1* or ΔIΔV-*IRE1*. We used the PCR primer set 5′-CAAGCGATTTAGATTTTAGAATTG-3′ and 5′-AATACTCCAGTCTCTATAATTCGA-3′ to check the ΔV-mutant genotype to obtain the relative abundance of ΔV-*IRE1* cells (%) from mixed cultures of cells expressing wild-type *IRE1* cells or ΔV-*IRE1*.

### RNA analysis

After extraction from cells as described by Kimata et al. (Kimata et al., 2003), total RNA samples were used as templates for reverse transcription (RT)-PCR amplification of the *HAC1* mRNA species, which was then separated by electrophoresis (Promlek et al., 2011; Ishiwata-Kimata et al., 2013). DNA fluorescence images of the resulting gels were captured and quantitatively analyzed using the LAS-4000 cooled CCD camera system. The resulting data were used to calculate the *HAC1* mRNA splicing efficiency (%) from the formula 100×[*HAC1*^i^ band signal/(*HAC1*^i^ band signal+*HAC1*^u^ band signal)].

### Fluorescent-protein tagging and localization of Ire1

An mNeonGreen-coding sequence (Shaner et al., 2013) with a codon usage optimized for yeast was inserted into the Ire1-coding region of pRS313-IRE1 and its ΔIΔV mutant. See Aragón et al. (Aragón et al., 2009) for the insertion position on the *IRE1* gene. After transformation of the KMY1015 strain with the resulting plasmid, mNeonGreen fluorescent images of cells were captured using the Delta Vision Elite microscopy system (Applied Precision) with the GFP excitation/emission filter set.

### UPRE-lacZ reporter assay

The KMY1015 strain carrying pCZY1 or the KMY1516 strain were transformed with *IRE1* gene plasmids and checked for cellular β-galactosidase, as described previously (Kimata et al., 2003). Data from multiple (more than three) independent transformant clones were used to calculate the means and standard deviations.

### Yeast two-hybrid assay

Y2HGold cells transformed with a bait plasmid and Y871 cells transformed with a prey plasmid were mated through mixed culturing, as described in the manufacturer's instructions (Clontech). The resulting cultures were serially tenfold diluted and spotted onto agar plates, incubated for 2–4 days at 30°C and photographed. The agar plates were synthetic dextrose supplemented with the –Leu/–Trp Dropout supplement (Yeast Protocol Handbook, Clontech) for selecting mated cells (the growth control plates) and those containing 125 ng/ml aureobasidin A for checking the two-hybrid interaction. In the experiment shown in Fig. 7A, agar plates for checking the two-hybrid interaction did not contain histidine, because the two-hybrid interaction provides the tester diploid cells with histidine prototrophy as well as with aureobasidin A resistance.

### *In vitro* competition assay for peptide binding to the CSSR

The His-tagged MBP–CSSR protein was expressed in *E. coli* and purified using a nickel affinity column and elution buffer A [50 mM HEPES pH 8.0, 100 mM KCl, 5 mM MgCl_2_, 200 mM imidazole and 10% (v/v) glycerol] as reported previously (Kimata et al., 2007). A 5-carboxyfluorescein (5-FAM)-tagged peptide (ΔEspP–FAM; Gardner and Walter, 2011) was chemically synthesized by GL Biochem (supplementary material Table S6; Shanghai, China). The untagged competitor peptides NH_2_-KKKA-[10 amino acids of Segment 2, 3, 4, 5 or 6]-AAKKKK-COOH were chemically synthesized by Sigma-Aldrich (supplementary material Table S6; the peptide screening value pack). The lysine clusters on the competitor peptides were expected to serve as solubilization tags. Segment 1 was not used in this assay because of its low chemical synthesis yield. After incubating the mixture in elution buffer A for 30 min, fluorescence anisotropy was measured using the BEACON™2000 fluorescence polarization reader (Invitrogen) at 25°C.

### Mammalian cell culturing and manipulation

NIH3T3 cells were cultured in Dulbecco's modified Eagle's medium containing 10% fetal bovine serum (37°C, 10% CO_2_). Plasmids were transfected into NIH3T3 cells by using Lipofectamine LTX with PLUS Reagent (Invitrogen) according to the manufacturer's protocol. Standard RIPA buffer was used for preparation of cell lysates.

### Antibodies

For western blot analysis, we used 12CA5 anti-HA mouse monoclonal antibody (Roche), 9E10 anti-c-Myc mouse monoclonal antibody (Boehringer Mannheim) and anti-eIF2α[pS^52^] rabbit polyclonal antibody (BioSource). As the secondary antibodies, we used goat anti-mouse IgG horseradish peroxidase (HRP)-linked antibody (Jackson ImmunoResearch) and goat anti-rabbit IgG HRP-linked antibody (Dako).

## 

**Table 1.**
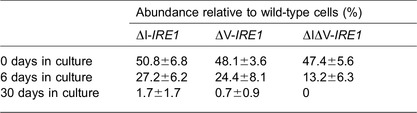
Growth retardation of unstressed yeast cells due to the ΔI, the ΔV or the ΔIΔV mutations

## Supplementary Material

Supplementary Material

## References

[b01] Aragón T, van Anken E, Pincus D, Serafimova IM, Korennykh AV, Rubio CA, Walter P (2009). Messenger RNA targeting to endoplasmic reticulum stress signalling sites.. Nature.

[b02] Berger JM, Gamblin SJ, Harrison SC, Wang JC (1996). Structure and mechanism of DNA topoisomerase II.. Nature.

[b03] Bertolotti A, Zhang Y, Hendershot LM, Harding HP, Ron D (2000). Dynamic interaction of BiP and ER stress transducers in the unfolded-protein response.. Nat. Cell Biol..

[b04] Chawla A, Chakrabarti S, Ghosh G, Niwa M (2011). Attenuation of yeast UPR is essential for survival and is mediated by IRE1 kinase.. J. Cell Biol..

[b05] Cox JS, Walter P (1996). A novel mechanism for regulating activity of a transcription factor that controls the unfolded protein response.. Cell.

[b06] Credle JJ, Finer-Moore JS, Papa FR, Stroud RM, Walter P (2005). On the mechanism of sensing unfolded protein in the endoplasmic reticulum.. Proc. Natl. Acad. Sci. USA.

[b07] Gardner BM, Walter P (2011). Unfolded proteins are Ire1-activating ligands that directly induce the unfolded protein response.. Science.

[b08] Harding HP, Zhang Y, Ron D (1999). Protein translation and folding are coupled by an endoplasmic-reticulum-resident kinase.. Nature.

[b09] Ishida T, Kinoshita K (2007). PrDOS: prediction of disordered protein regions from amino acid sequence.. Nucleic Acids Res..

[b10] Ishiwata-Kimata Y, Promlek T, Kohno K, Kimata Y (2013). BiP-bound and nonclustered mode of Ire1 evokes a weak but sustained unfolded protein response.. Genes Cells.

[b11] Iwawaki T, Akai R, Yamanaka S, Kohno K (2009). Function of IRE1 alpha in the placenta is essential for placental development and embryonic viability.. Proc. Natl. Acad. Sci. USA.

[b12] Iwawaki T, Akai R, Kohno K (2010). IRE1α disruption causes histological abnormality of exocrine tissues, increase of blood glucose level, and decrease of serum immunoglobulin level.. PLoS ONE.

[b13] Kimata Y, Kohno K (2011). Endoplasmic reticulum stress-sensing mechanisms in yeast and mammalian cells.. Curr. Opin. Cell Biol..

[b14] Kimata Y, Kimata YI, Shimizu Y, Abe H, Farcasanu IC, Takeuchi M, Rose MD, Kohno K (2003). Genetic evidence for a role of BiP/Kar2 that regulates Ire1 in response to accumulation of unfolded proteins.. Mol. Biol. Cell.

[b15] Kimata Y, Oikawa D, Shimizu Y, Ishiwata-Kimata Y, Kohno K (2004). A role for BiP as an adjustor for the endoplasmic reticulum stress-sensing protein Ire1.. J. Cell Biol..

[b16] Kimata Y, Ishiwata-Kimata Y, Yamada S, Kohno K (2006). Yeast unfolded protein response pathway regulates expression of genes for anti-oxidative stress and for cell surface proteins.. Genes Cells.

[b17] Kimata Y, Ishiwata-Kimata Y, Ito T, Hirata A, Suzuki T, Oikawa D, Takeuchi M, Kohno K (2007). Two regulatory steps of ER-stress sensor Ire1 involving its cluster formation and interaction with unfolded proteins.. J. Cell Biol..

[b18] Korennykh AV, Egea PF, Korostelev AA, Finer-Moore J, Zhang C, Shokat KM, Stroud RM, Walter P (2009). The unfolded protein response signals through high-order assembly of Ire1.. Nature.

[b19] Li H, Korennykh AV, Behrman SL, Walter P (2010). Mammalian endoplasmic reticulum stress sensor IRE1 signals by dynamic clustering.. Proc. Natl. Acad. Sci. USA.

[b20] Liu CY, Schröder M, Kaufman RJ (2000). Ligand-independent dimerization activates the stress response kinases IRE1 and PERK in the lumen of the endoplasmic reticulum.. J. Biol. Chem..

[b21] Ma Y, Shimizu Y, Mann MJ, Jin Y, Hendershot LM (2010). Plasma cell differentiation initiates a limited ER stress response by specifically suppressing the PERK-dependent branch of the unfolded protein response.. Cell Stress Chaperones.

[b22] Mori K (2009). Signalling pathways in the unfolded protein response: development from yeast to mammals.. J. Biochem..

[b23] Mori K, Sant A, Kohno K, Normington K, Gething MJ, Sambrook JF (1992). A 22 bp cis-acting element is necessary and sufficient for the induction of the yeast KAR2 (BiP) gene by unfolded proteins.. EMBO J..

[b24] Mori K, Kawahara T, Yoshida H, Yanagi H, Yura T (1996). Signalling from endoplasmic reticulum to nucleus: transcription factor with a basic-leucine zipper motif is required for the unfolded protein-response pathway.. Genes Cells.

[b25] Mori K, Ogawa N, Kawahara T, Yanagi H, Yura T (2000). mRNA splicing-mediated C-terminal replacement of transcription factor Hac1p is required for efficient activation of the unfolded protein response.. Proc. Natl. Acad. Sci. USA.

[b26] Oikawa D, Kimata Y, Takeuchi M, Kohno K (2005). An essential dimer-forming subregion of the endoplasmic reticulum stress sensor Ire1.. Biochem. J..

[b27] Oikawa D, Kimata Y, Kohno K (2007). Self-association and BiP dissociation are not sufficient for activation of the ER stress sensor Ire1.. J. Cell Sci..

[b28] Pincus D, Chevalier MW, Aragón T, van Anken E, Vidal SE, El-Samad H, Walter P (2010). BiP binding to the ER-stress sensor Ire1 tunes the homeostatic behavior of the unfolded protein response.. PLoS Biol..

[b29] Promlek T, Ishiwata-Kimata Y, Shido M, Sakuramoto M, Kohno K, Kimata Y (2011). Membrane aberrancy and unfolded proteins activate the endoplasmic reticulum stress sensor Ire1 in different ways.. Mol. Biol. Cell.

[b30] Rosenbaum JC, Fredrickson EK, Oeser ML, Garrett-Engele CM, Locke MN, Richardson LA, Nelson ZW, Hetrick ED, Milac TI, Gottschling DE (2011). Disorder targets misorder in nuclear quality control degradation: a disordered ubiquitin ligase directly recognizes its misfolded substrates.. Mol. Cell.

[b31] Rubio C, Pincus D, Korennykh A, Schuck S, El-Samad H, Walter P (2011). Homeostatic adaptation to endoplasmic reticulum stress depends on Ire1 kinase activity.. J. Cell Biol..

[b32] Shaner NC, Lambert GG, Chammas A, Ni Y, Cranfill PJ, Baird MA, Sell BR, Allen JR, Day RN, Israelsson M (2013). A bright monomeric green fluorescent protein derived from Branchiostoma lanceolatum.. Nat. Methods.

[b33] Sikorski RS, Hieter P (1989). A system of shuttle vectors and yeast host strains designed for efficient manipulation of DNA in Saccharomyces cerevisiae.. Genetics.

[b34] Travers KJ, Patil CK, Wodicka L, Lockhart DJ, Weissman JS, Walter P (2000). Functional and genomic analyses reveal an essential coordination between the unfolded protein response and ER-associated degradation.. Cell.

[b35] Tsuru A, Fujimoto N, Takahashi S, Saito M, Nakamura D, Iwano M, Iwawaki T, Kadokura H, Ron D, Kohno K (2013). Negative feedback by IRE1β optimizes mucin production in goblet cells.. Proc. Natl. Acad. Sci. USA.

[b36] Walter P, Ron D (2011). The unfolded protein response: from stress pathway to homeostatic regulation.. Science.

