# Supplementary material for: Tight regulation of the unfolded protein sensor Ire1 by its intramolecularly antagonizing subdomain
Source: J Cell Sci. 2015 May 1;128(9):1762–72. doi: 10.1242/jcs.164111 (PMC4432228; doi:10.1242/jcs.164111)
Supplement: Supplementary Material [file supp_128_9_1762__index.html]

Tight regulation of the unfolded protein sensor Ire1 by its intramolecularly antagonizing subdomain — Supplementary Material 

# Tight regulation of the unfolded protein sensor Ire1 by its intramolecularly antagonizing subdomain

## JCS164111 Supplementary Material

**Files in this Data Supplement:**

- **Supplementary Material**
