# Supplementary material for: Tight regulation of the unfolded protein sensor Ire1 by its intramolecularly antagonizing subdomain
Source: J Cell Sci. 2015 May 1;128(9):1762–72. doi: 10.1242/jcs.164111 (PMC4432228; doi:10.1242/jcs.164111)
Supplement: Supplementary Material [file supp_128.9.1762_JCS164111.pdf]

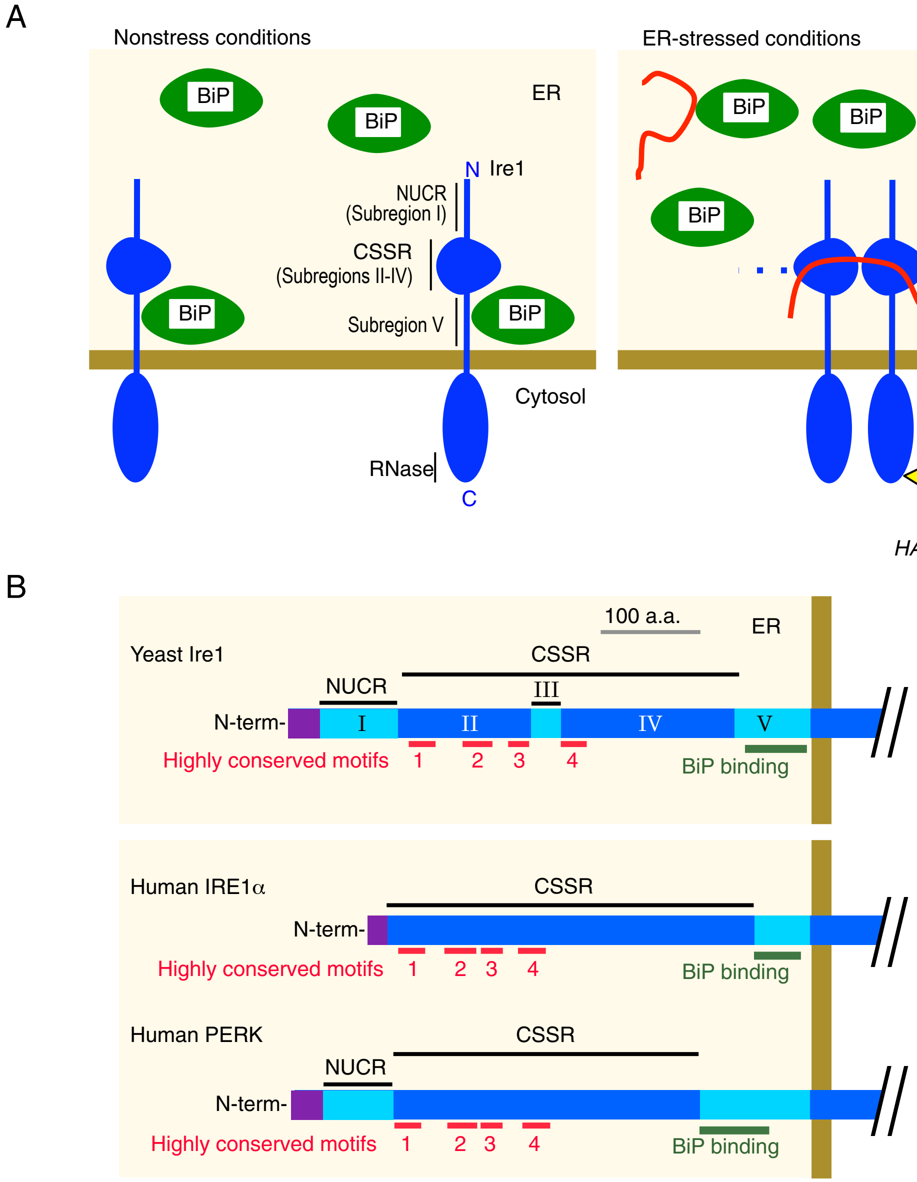

Figure S1 Structure and regulation of the Ire1 family proteins

A, Regulation of yeast Ire1 by BiP association/dissociation and by the direct interaction of unfolded proteins is schematically represented. See Kimata et al. [J. Cell Biol., Vol. 167, 445–456 (2004)] for partitioning of the Ire1 luminal domain into Subregions I–V. Subregions II–IV are tightly folded and correspond to the CSSR (a.a. 112–454). Upon ER stress, Ire1 is clustered and activated by dissociation of BiP from Subregion V (a.a. 455–524) and interaction of unfolded proteins with the CSSR [Kimata and Kohno, Curr. Opin. Cell. Biol., Vol. 23, 135–142 (2011)]. Meanwhile, the regulatory function of Subregion I (a.a. 32–111) has been obscure. **B,** Structural comparison of the luminal domains of Ire1-family proteins is schematically represented. The CSSR show low but significant homology, whereas the amino-acid sequences of the NUCRs and the BiP-binding sites are less conserved. Highly conserved motifs 1–4 observed on the CSSRs [Liu et al., J. Biol. Chem., Vol. 275, 24881–24885 (2000)] are marked with red lines. Modified from Kimata and Kohno [Curr. Opin. Cell. Biol., Vol. 23, 135–142 (2011)].

Figure S2

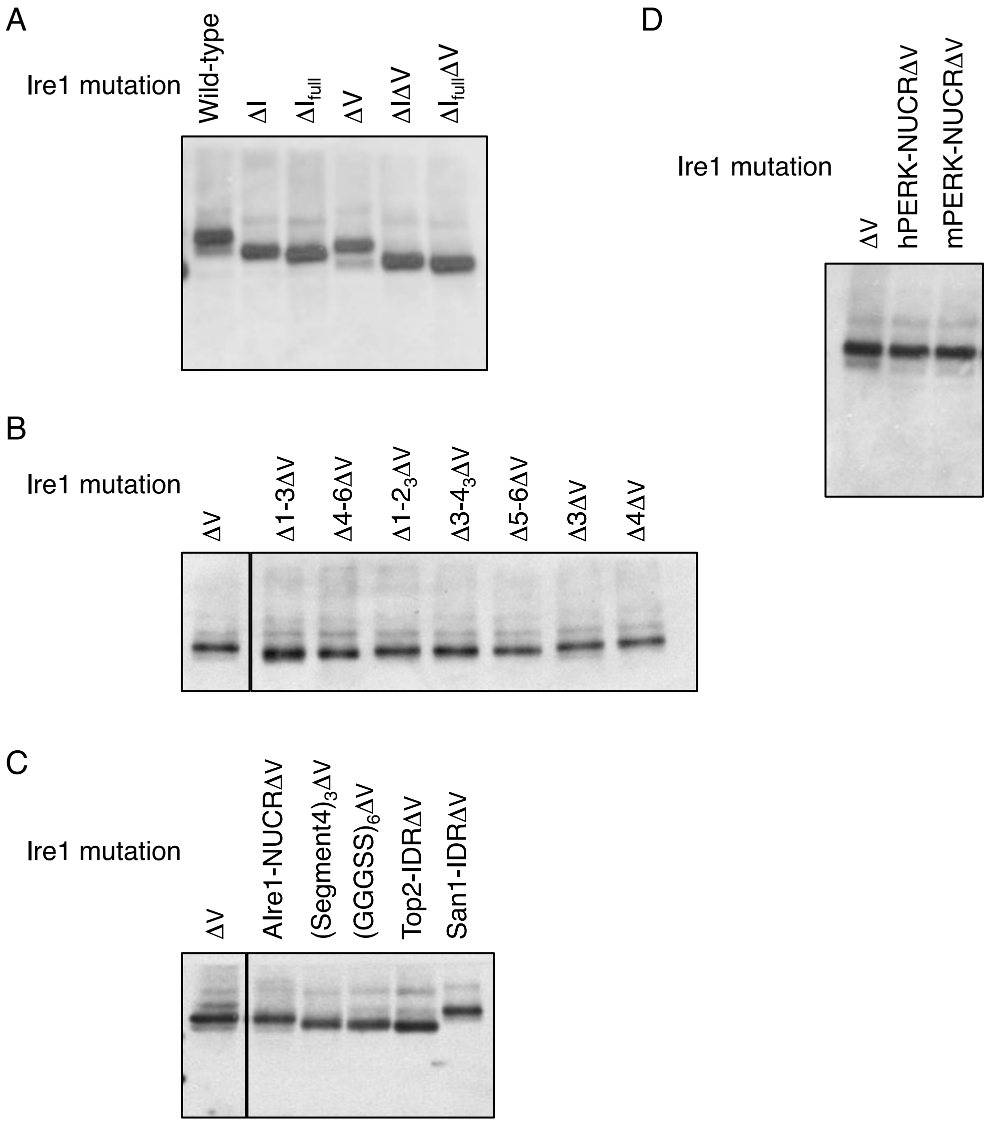

<u>Figure S2</u> <u>Cellular expression levels of Ire1 and its mutants</u>
KMY1015 *ire1Δ* cells were cultured under unstressed conditions after transformation with pRS315-IRE1-HA [a yeast single-copy plasmid carrying the wild-type (WT) *IRE1* gene modified to harbor the C-terminal hemagglutinin (HA) epitope; Kimata et al., J. Cell Biol., Vol. 167, 445–456 (2004)] or its mutants. Cell lysates equivalent to 10 μg total protein were analyzed by anti-HA Western blotting as described in Kimata et al. [J. Cell Biol., Vol. 167, 445–456 (2004)]. The two panels in B are from the same gel. The two panels in C are also from the same gel.

Figure S3

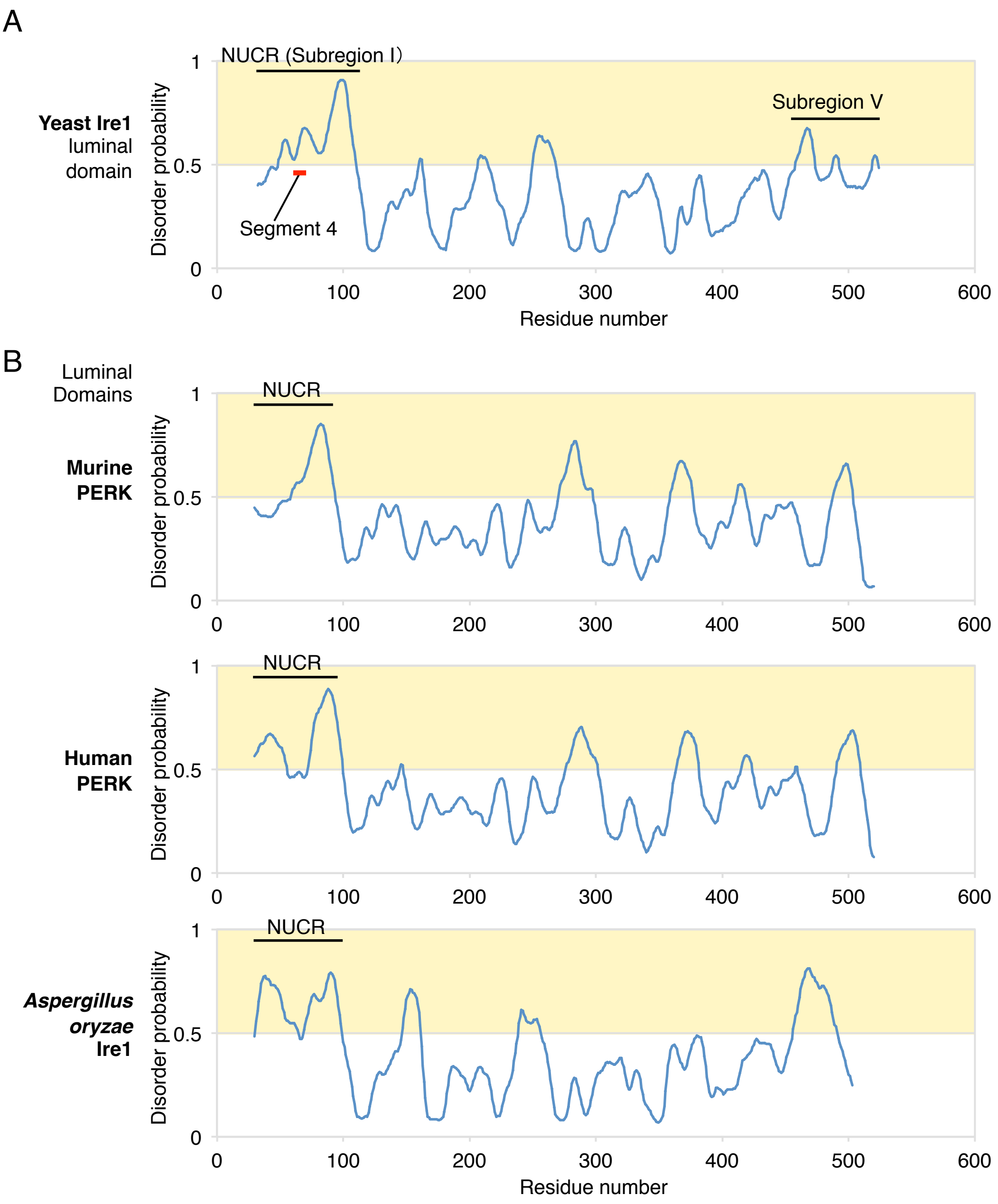

Figure S3 Protein structure prediction of the luminal regions of Ire1 family proteins carrying the NUCRs
Amino acid sequences of the luminal regions of yeast Ire1 (A) and others (B) were inputted into PrDOS (http://prdos.hgc.jp/cgi-bin/top.cgi).

Figure S4

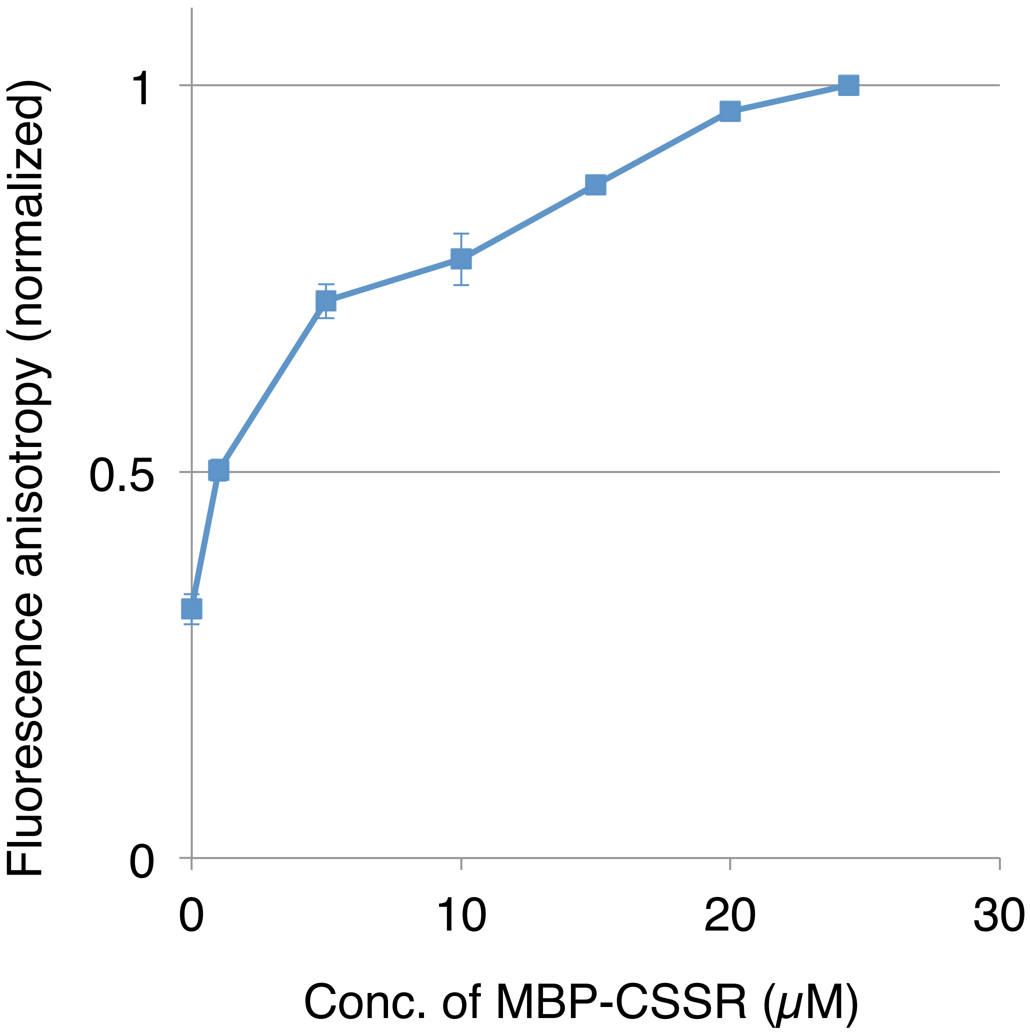

Figure S4 *In vitro* association of the CSSR with a fluorescently labeled peptide

The ΔEspP-FAM fluorescently labeled peptide (10 μM final) was mixed with MBP-CSSR, and fluorescence anisotropy was measured. The resulting values from triplicate assays (means plus standard deviations) are normalized against that for the maximum value.

Supplementary tables Amino-acid sequences of the Subregion-I mutations, the two-hybrid preys and baits and the synthetic peptides employed in this study.

Table S1. The Subregion-I 60-a.a. portion of wild-type yeast Ire1

TSRRQIVEDEVASTKKLNFNYGVDKNINSPIPAPRTTEGLPNMKLSSYPTPNLLNTADNR

Segments 1, 3 and 5 are blue-colored. Segment 4 is orange-colored. Segments 2 and 6 are brown-colored.

Table S2. The partial-deletion mutations of the Subregion-I 60-a.a. portion

Δ1-3	IPAPRTTEGLPNMKLSSYPTPNLLNTADNR
Δ4-6	TSRRQIVEDEVASTKKLNFNYGVDKNINSP
Δ1-2	YGVDKNINSPIPAPRTTEGLPNMKLSSYPTPNLLNTADNR
Δ3-4	TSRRQIVEDEVASTKKLNFNPNMKLSSYPTPNLLNTADNR
Δ5-6	TSRRQIVEDEVASTKKLNFNYGVDKNINSPIPAPRTTEGL
Δ3	TSRRQIVEDEVASTKKLNFNIPAPRTTEGLPNMKLSSYPTPNLLNTADNR
Δ4	TSRRQIVEDEVASTKKLNFNYGVDKNINSPPNMKLSSYPTPNLLNTADNR

Segments 2, 4 and 6 are blue-colored. Segment 4 is orange-colored. Segments 2 and 6 are brown-colored.

Table S3. The peptides substituted on the Subregion-I 60-a.a. portion of ΔV Ire1

San1-IDR	TSVPTIGNASSGEQMLSRTGFFLVPQNGQPLHNPVRLPPNDSDRNGVNGPSSTTQNPPTR
Top2-IDR	TSDKDYIDLAFSKKKADDRKEWLRQYEPTR
$(GGGSS)_6$	TSGGGSSGGGSSGGGSSGGGSSGGGSSGGGSSTR
$(Segment 4)_3$	TSIPAPRTTEGLIPAPRTTEGLIPAPRTTEGLTR
mPERK-NUCR	TSVAPARSLLAPASETVFGLGAAAAPTSAARVPAVATAEVTVEDAEALPAAAGETR
hPERK-NUCR	TSGRARGLPAPTAEAAFGLGAAAAPTSATRVPAAGAVAAAEVTVEDAEALPAAATR
AIre1-NUCR	TSQQQPEHHDLPSTLSVPLGSTGHAVGKDLYTPLNVKSTDASALATMALAGPGRTR
mPERK-NUCR anterior 26 a.a.	TSVAPARSLLAPASETVFGLGAAAAPTSTR
mPERK-NUCR posterior 26 a.a.	TSAARVPAVATAEVTVEDAEALPAAAGETR
hPERK-NUCR anterior 26 a.a.	TSGRARGLPAPTAEAAFGLGAAAAPTSATR
hPERK-NUCR posterior 26 a.a.	TSTRVPAAGAVAAAEVTVEDAEALPAAATR
AIre1-NUCR anterior 26 a.a.	TSQQQPEHHDLPSTLSVPLGSTGHAVGKTR

AIre1-NUCR posterior 26 a.a.	TSDLYTPLNVKSTDASALATMALAGPGRTR

The amino-acid residues corresponding to the artificially added restriction sites (*Spe*I (TS) and *Mlu*I (TR)) are green-colored. Segment 4 is orange-colored.

Table S4. The two-hybrid bait peptides

Sub-I (the Subregion-I 60-a.a. portion)	EFMSRRQIVEDEVASTKKLNFNYGVDKNINSPIPAPRTT<br>EGLPNMKLSSYPTPNLLNTADNRGS
Seg4 (six tandem repeats of Segment 4 (IPAPRTTEGL))	EFMIPAPRTTEGLIPAPRTTEGLIPAPRTTEGLIDIPAP<br>RTTEGLIPAPRTTEGLIPAPRTTGS
GGGSS (tandem repeats of GGGSS)	EFMSGGGSSGGGSSGGGSSGGGSSGGGSSGGIDGGGGSS<br>GGGSSGGGSSGGGSSGGGSSGGGGS
Seg1-3 (two tandem repeats of Segment 1 to 3)	EFMTSRRQIVEDEVASTKKLNFNYGVDKNINSPIDTSRR<br>QIVEDEVASTKKLNFNYGVDKNINSPGS
Seg4-6 (two tandem repeats of Segment 4 to 6)	EFMIPAPRTTEGLPNMKLSSYPTPNLLNTADNRIDIPAP<br>RTTEGLPNMKLSSYPTPNLLNTADNRGS
hPERK (the NUCR of human PERK)	EFMGRARGLPAPTAEAAFGLGAAAAPTSATRVPAAGAVA<br>AAEVTVEDAEALPAAAGEQEPRGGS
mPERK (the NUCR of mouse PERK)	EFMVAPARSLLAPASETVFGLGAAAAPTSAARVPAVATA<br>EVTVEDAEALPAAAGEPESRATEGS
Top2 (two tandem repeats of the Top2 IDR)	EFMDKDYIDLAFSKKKADDRKEWLRQYEPIDDKDYIDLA<br>FSKKKADDRKEWLRQYEPGS
San1 (the San1 IDR)	EFMVPTIGNASSGEQMLSRTGFFLVPQNGQPLHNPVRLP<br>PNDSDRNGVNGPSSTTQNPPSNSGS
CSSR	PWMSLNELSLSDILIAADVEGGLHAVDRRNGHIIWSIEP<br>ENFQPLIEIQEPSRLETYETLIIEPFGDGNIYYFNAHQG<br>LQKLPLSIRQLVSTSPLHLKTNIVVNDSGKIVEDEKVYT<br>GSMRTIMYTINMLNGEIISAFGPGSKNGYFGSQSVDCSP<br>EEKIKLQECENMIVIGKTIFELGIHSYDGASYNVTYSTW<br>QQNVLDVPLALQNTFSKDGMCIAPFRDKSLLASDLDFRI<br>ARWVSPTFPGIIVGLFDVFNDLRTNENILVPHPFNPGDH<br>ESISSNKVYLDQTSNLSWFALSSQNFPSLVESAPISRYA<br>SSDRWRVSSIFEDETLFKNAIMGVHQIYNNEYDGS
Sub-I-CSSR (Subregion 1 to 4)	PWMSRRQIVEDEVASTKKLNFNYGVDKNINSPIPAPRTT<br>EGLPNMKLSSYPTPNLLNTADNRRANKKGRRAANSISVP<br>YLENRSLNELSLSDILIAADVEGGLHAVDRRNGHIIWSI<br>EPENFQPLIEIQEPSRLETYETLIIEPFGDGNIYYFNAH<br>QGLQKLPLSIRQLVSTSPLHLKTNIVVNDSGKIVEDEKV<br>YTGSMRTIMYTINMLNGEIISAFGPGSKNGYFGSQSVDC<br>SPEEKIKLQECENMIVIGKTIFELGIHSYDGASYNVTYS<br>TWQQNVLDVPLALQNTFSKDGMCIAPFRDKSLLASDLDF<br>RIARWVSPTFPGIIVGLFDVFNDLRTNENILVPHPFNPG<br>DHESISSNKVYLDQTSNLSWFALSSQNFPSLVESAPISR<br>YASSDRWRVSSIFEDETLFKNAIMGVHQIYNNEYDGS
The ΔIII mutant of Sub-I-CSSR	PWMSRRQIVEDEVASTKKLNFNYGVDKNINSPIPAPRTT<br>EGLPNMKLSSYPTPNLLNTADNRRANKKGRRAANSISVP<br>YLENRSLNELSLSDILIAADVEGGLHAVDRRNGHIIWSI<br>EPENFQPLIEIQEPSRLETYETLIIEPFGDGNIYYFNAH<br>QGLQKLPLSIRQLVSTSPLHLKTNIVVNDSGKIVEDEKV<br>YTGSMRTIMYTINMLNGEIISAFGPGSKECENMIVIGKT<br>IFELGIHSYDGASYNVTYSTWQQNVLDVPLALQNTFSKD<br>GMCIAPFRDKSLLASDLDFRIARWVSPTFPGIIVGLFDV<br>FNDLRTNENILVPHPFNPGDHESISSNKVYLDQTSNLSW<br>FALSSQNFPSLVESAPISRYASSDRWRVSSIFEDETLFK

	NAIMGVHQIYNNEYDGS
The MFY (M229A / F285A / Y301A) mutant of Sub-I-CSSR	PWMSRRQIVEDEVASTKKLNFNYGVDKNINSPIPAPRTT<br>EGLPNMKLSSYPTPNLLNTADNRRANKKGRRAANSISVP<br>YLENRSLNELSLSDILIAADVEGGLHAVDRRNGHIIWSI<br>EPENFQPLIEIQEPSRLETYETLIIEPFGDGNIYYFNAH<br>QGLQKLPLSIRQLVSTSPLHLKTNIVVNDSGKIVEDEKV<br>YTGSARTIMYTINMLNGEIISAFGPGSKNGYFGSQSVDC<br>SPEEKIKLQECENMIVIGKTIAELGIHSYDGASYNVTAS<br>TWQQNVLDVPLALQNTFSKDGMCIAPFRDKSLLASDLDF<br>RIARWVSPTFPGIIVGLFDVFNDLRTNENILVPHPFNPG<br>DHESISSNKVYLDQTSNLSWFALSSQNFPSLVESAPISR<br>YASSDRWRVSSIFEDETLFKNAIMGVHQIYNNEYDGS

The amino-acid residues corresponding to the artificially added restriction sites (*Eco*RI (EF), *Bam*HI (GS), *Cla*I (ID) and *Nco*I (PW)) are green-colored. Segments 1, 3 and 5 are blue-colored. Segment 4 is orange-colored. Segments 2 and 6 are brown-colored. The CSSR is purple-colored. The mutation sites of the MFY mutation are yellow-colored.

Table S5. The two-hybrid prey peptides

CSSR	GSMRSLNELSLSDILIAADVEGGLHAVDRRNGHIIWSIEPENFQPLIEIQ<br>EPSRLETYETLIIEPFGDGNIYYFNAHQGLQKLPLSIRQLVSTSPLHLKT<br>NIVVNDSGKIVEDEKVYTGSMRTIMYTINMLNGEIISAFGPGSKNGYFGS<br>QSVDCSPEEKIKLQECENMIVIGKTIFELGIHSYDGASYNVTYSTWQQNV<br>LDVPLALQNTFSKDGMCIAPFRDKSLLASDLDFRIARWVSPTFPGIIVGL<br>FDVFNDLRTNENILVPHPFNPGDHESISSNKVYLDQTSNLSWFALSSQNF<br>PSLVESAPISRYASSDRWRVSSIFEDETLFKNAIMGVHQIYNNEYDter
The ΔIII mutant of CSSR	GSMRSLNELSLSDILIAADVEGGLHAVDRRNGHIIWSIEPENFQPLIEIQ<br>EPSRLETYETLIIEPFGDGNIYYFNAHQGLQKLPLSIRQLVSTSPLHLKT<br>NIVVNDSGKIVEDEKVYTGSMRTIMYTINMLNGEIISAFGPGSKECENMI<br>VIGKTIFELGIHSYDGASYNVTYSTWQQNVLDVPLALQNTFSKDGMCIAP<br>FRDKSLLASDLDFRIARWVSPTFPGIIVGLFDVFNDLRTNENILVPHPFN<br>PGDHESISSNKVYLDQTSNLSWFALSSQNFPSLVESAPISRYASSDRWRV<br>SSIFEDETLFKNAIMGVHQIYNNEYDter
The MFY mutant of CSSR	GSMRSLNELSLSDILIAADVEGGLHAVDRRNGHIIWSIEPENFQPLIEIQ<br>EPSRLETYETLIIEPFGDGNIYYFNAHQGLQKLPLSIRQLVSTSPLHLKT<br>NIVVNDSGKIVEDEKVYTGSARTIMYTINMLNGEIISAFGPGSKNGYFGS<br>QSVDCSPEEKIKLQECENMIVIGKTIAELGIHSYDGASYNVTASTWQQNV<br>LDVPLALQNTFSKDGMCIAPFRDKSLLASDLDFRIARWVSPTFPGIIVGL<br>FDVFNDLRTNENILVPHPFNPGDHESISSNKVYLDQTSNLSWFALSSQNF<br>PSLVESAPISRYASSDRWRVSSIFEDETLFKNAIMGVHQIYNNEYDter

The amino-acid residues corresponding to the artificially added restriction site (*Bam*HI (GS)) are green-colored. The CSSR is purple-colored. The mutation sites of the MFY mutation are yellow-colored.

Table S6. The chemically synthesized peptides

Fluorescently labeled peptide	
ΔEspP-FAM	MKKHKRILALCFLGLLQSSYSFAK-[5-FAM]
Competitor peptides	
Segment 2	KKKAVASTKKLNFNAAKKKK
Segment 3	KKKAYGVDKNINSPAAKKKK
Segment 4	KKKAIPAPRTTEGLAAKKKK
Segment 5	KKKAPNMKLSSYPTAAKKKK
Segment 6	KKKAPNLLNTADNRAAKKKK

Segments 2 and 6 are brown-colored. Segments 3 and 5 are blue-colored. Segment 4 is orange-colored.
